# A multidisciplinary primary prevention intervention to increase adherence to the Mediterranean diet: a pilot study

**DOI:** 10.1186/s12889-023-16893-0

**Published:** 2023-10-19

**Authors:** Silvia De Marco, Eleonora Marziali, Lorenza Nachira, Paola Arcaro, Leonardo Villani, Valerio Galasso, Stefania Bruno, Patrizia Laurenti

**Affiliations:** 1 Faculty of Medicine and Surgery, Università Cattolica del Sacro Cuore, Rome, Italy; 2 Department of Life Science and Public Health, Università Cattolica del Sacro Cuore, Rome, Italy; 3 DiagnostiCare ONLUS, Rome, Italy; 4 Women, Children and Public Health Sciences Department, Fondazione Policlinico Universitario A. Gemelli IRCCS, Rome, Italy

**Keywords:** Educational interventions, Primary prevention, Multidisciplinary intervention, Cardiovascular diseases, Mediterranean diet

## Abstract

**Background:**

The role of the Mediterranean Diet (MD) in reducing cardiovascular (CV) risk is widely demonstrated and many studies have shown the effectiveness of educational interventions in primary prevention. This study aimed to evaluate the impact of a multidisciplinary educational intervention, that included nutritional, psychological and physical activity coaching, on adherence to MD and on CV risk.

**Methods:**

In a Roman neighborhood, general practitioners enrolled 41 subjects to take part in the educational intervention from November 2018 (T_0_) to November 2019 (T_1_). Participants’ anthropometric measures, haematochemical parameters and CV risk score were assessed before and after the intervention. Furthermore, their adherence to MD was evaluated through the analysis of food frequency questionnaires using Medi-Lite.

**Results:**

The study found a significant reduction of 2.5 points in individual CV risk score, and an increase of 2.5 point in adherence to the MD. The stratification by gender showed statistically significant decreases in weight of 1.16 kg, BMI of 0.47, LDL cholesterol of 14.00 mg/dL, and individual CV risk score of 1.16 points among female participants.

**Conclusions:**

These results show that a multidisciplinary educational intervention model including the adoption of MD could be an effective strategy in Public Health for CV primary prevention and improvement of people’s lifestyles.

## Background

Cardiovascular diseases (CVDs) are a group of disorders of the heart and blood vessels, representing the leading cause of mortality and disability worldwide [1–3]. Over the past 40 years, the prevalence of these diseases has doubled, and the number of deaths and disability-adjusted life years (DALYs) has increased dramatically, making CVDs a major Public Health issue [4, 5]. As recently as 2019, CVDs caused 18.6 million deaths, the 30% of them occurred prematurely in people under the age of 70 [1]. A similar trend of increasing prevalence, mortality, and DALYs is also observed in Italy, [6] such that in 2018 CVDs caused 220.456 deaths representing the main three causes of death (27.1% of all deaths in 2018) [1]. There are many risk factors affecting CVDs related to lifestyle, such as unbalanced diet, low physical activity, obesity, diabetes, high levels of blood cholesterol and blood pressure (BP), smoking habits [7]. Indeed, many studies have evaluated the correlation between balanced diet and CV risk reduction [8, 9]. Specifically, there is evidence on the role of the Mediterranean Diet (MD) as a preventive factor for various clinical conditions [10, 11]. such as CVDs (hypertension, arteriosclerosis, stroke, myocardial infarction), overweight and obesity, metabolic disorders (type 2 diabetes mellitus, hypercholesterolemia or other dyslipidaemias) and cancer (stomach, esophagus, colon, rectum and breast) [12–15]. As reported by Cowell in a systematic review, the adoption of the MD can reduce systolic blood pressure (SBP) and diastolic blood pressure (DBP) [16]. These health outcomes are related to the composition, distribution and frequency of consumption of Mediterranean food, in particular the intake of fruit, vegetables, cereals, olive oil and very low consumption of alcohol and highly sweetened food [17, 18].

In Italy, in order to decrease the national health and economic burden of metabolic and cardiovascular diseases and to improve the support for healthy nutrition, research Institutions, such as the “Council for Agricultural Research and Agricultural Economics Analysis” (CREA – Italian acronym) and the “Italian Society of Human Nutrition” (SINU – Italian acronym), have worked on many documents and position papers about nutrition, Public Health and educational programmes to promote healthy diet habits among the population [19–22]. These could be considered as prevention models when applied to the population within a study. Indeed, several studies showed the effectiveness of educational interventions as tools in primary prevention on a population level [23–25].

In this context, the aim of this study is (I) to evaluate the impact of a multidisciplinary educational intervention on cardiovascular risk factors, assessing the variation of anthropometric and haematochemical parameters, blood pressure and individual cardiovascular risk score [26, 27]. and (II) to evaluate the adherence variation to the MD, through the analysis of the Medi-Lite questionnaire filled out before and after the intervention.

## Methods

### Study design and sample

This multidisciplinary pre-post observational study was conducted in the Roman neighborhood of Torresina, Italy, from November 2018 (Time 0 – T_0_) to November 2019 (Time 1 – T_1_). The study included several phases, as follows: sample selection, first examination, 1-year intervention, second examination, data collection and analysis. In particular, the participation in the study was offered to all patients between 35 and 69 years of age who attended the general practitioner’s (GP) office (4 GP were involved in the study) in September 2018 in the Torresina district of Rome. Considering the pilot nature of the study, a non-randomized convenience sampling method was performed, and therefore, the sample size was not calculated.

Participation was unpaid and informed consent had to be signed. Exclusion criteria included being pregnant, having experienced a previous cardiovascular event, having any kind of pathology, having SBP ≤ 90 mmHg or ≥ 200 mmHg, total cholesterol ≤ 130 mg/dL or ≥ 320 mg/dL, HDL cholesterol ≤ 20 mg/dL or ≥ 100 mg/dL. At the end of the sample recruitment in September 2018, the nurses of the non-profit organisation collected data such as weight, Body Mass Index (BMI), waist circumference, triglyceridemia, total cholesterol, LDL and HDL cholesterol, SBP and DBP.

In November 2018, the GPs analysed the data and performed the first medical examination. From November 2018 to November 2019 patients were involved in many educational activities: nutritional intervention, physical activity, and psychological sessions held respectively by a nutritionist, a coach and a personal trainer, and a psychologist belonging to the non-profit organisation. The first meeting for educational activities took place in 15 days starting from November 2018, the second meeting took place in 15 days starting from May 2019 and the third meeting took place at the end of the study in November 2019 where the different professionals gave advice to the patients on autonomous lifestyle management.

Finally, the anthropometric and haematochemical parameters were detected and analysed at the end of the educational activities, 12 months later (T_1_).

#### Psychological interventions

Psychological educational meetings were held by a psychologist who provided counseling and gave psychological support oriented toward smoking cessation or abstention and to strengthen the motivation for a healthy lifestyle.

#### Physical activity interventions

A physiotherapist, in collaboration with a motivational coach, dispensed advice and instructions on fitness trails aimed at healthy and regular physical activity.

#### Nutritional interventions

The nutritional intervention was held by a nutritionist of a non-profit organisation of the Torresina district. In the first meeting (November 2018), the nutritionist gave information about macronutrients and their partition in foods. It was recommended the consumption of carbohydrates, especially whole ones; water and hydration according to needs; salt was recommended for moderate use respecting the limit of 2–5 g/day; fruit and vegetables every day. The consumption of oily fish for the appropriate omega 3–6 ratio was recommended at least 3 times a week.

Then, in the following meetings, doubts were clarified, and the nutritionist counseled about grocery shopping and reading food labels. The outcome variables, particularly the MD adherence score calculated through the Medi-Lite questionnaire, [11, 28]. were recorded to monitor changes before and after the interventions. Data were collected through the administration of a not yet validated food frequency questionnaire (FFQ) created by the nutritionist of the project. However, a pilot survey had been previously conducted with a small sample of patients and it showed reliability. These FFQ expected to report the type, quantity and frequency of food consumed weekly for each meal (breakfast, lunch, dinner, and snacks) and the sensation experienced (hunger, thirst, boredom, satisfaction). The Medi-Lite questionnaire, that assessed the MD adherence score, is composed by nine sections for each food group: fruits, vegetables, legumes, cereals, fish, meat and salami, milk and dairy products, olive oil and alcohol. For each section, it was necessary to indicate the frequency which had its own corresponding score from 0 to 2. For each food group the score corresponded to a different weekly or daily frequency, and the sum of the different score gave the final MD adherence score [11, 28]. The changing of adherence to the MD is calculated by the increase of the Medi-Lite score.

#### Individual cardiovascular risk score

This parameter was used by the Italian National Institute of Health and in previous studies [26]. to predict the probability of death, stroke, heart attack or coronary heart injury within the next ten years. It considered some risk factors such as age, gender, total cholesterol, HDL cholesterol and systolic blood pressure, the use of antihypertensive drugs, the number of cigarettes per day for the smokers and the presence of diabetes. The score is represented by a percentage: low risk (< 3%), intermediate risk (3–20%), high risk (> 20%).

### Statistical analysis

Descriptive analyses were conducted to calculate gender, mean age, and occupational level. Inferential statistic tests were used to assess the change of variables following the intervention between T_0_ and T_1_ in terms of body weight, BMI, haematochemical parameters and the relative individual cardiovascular risk score. T-test and Wilcoxon test were used according to the distribution of continuous variables calculated by the Shapiro-Wilk test (normal distribution or not, respectively). P-values below 0.05 were considered statistically significant. All statistical analyses were performed using Stata software, version 14 (StataCorp LP, College Station, TX).

### Ethical approval

The study has been conducted in accordance with the GCP, with the Regulation (EU) 2016/679 on protection of natural people with the regard to the processing of personal data, as well as the free circulation of data and repealing Directive 95/46/EC (General Data Protection Regulation), and to all the legislation in force on this matter. Each participant has received the relative informed consent. The data relating to the patients and the investigations carried out have been anonymised by the GP (the only holder of the clinical data of his clients) through the assignment of a unique code to allow its aggregation and analysis, according to privacy protection criteria. The project was approved by the Ethics Committee of the Local Health Authority – ASL Roma 1, to which the General Practitioners refer c/o San Camillo Forlanini Hospital in Rome, with protocol number 1817/CE Lazio 1.

## Results

The study was made up of 41 enrolled individuals, 20 men and 21 women aged between 35 and 69 (mean age 55.4 years). In terms of educational level, most of them (56%) had completed secondary education (Table 1).

**Table 1:** Socio-demographic characteristics of the sample (N = 41)

Variables		n (%)
Gender	Male	20 (49)
	Female	21 (51)
Educational Level	Middle School	9 (22)
	High School	23 (56)
	University	9 (22)
Age	39–55	19 (46)
	56–72	22 (54)

Table 2 shows statistically significant results; body weight reduced by an average of 1.27 kg (95% Confidence Interval − 95%CI = 0.12–2.40; p = 0.03) and SBP decreased by an average of 13.22 mmHg (95%CI = 7.63–18.81; p < 0.0001) from T_0_ (November 2018) to T_1_ (November 2019).

**Table 2:** Differences in means and median, between T0 and T1, of the considered risk factors (waist circumference, weight, SBP, DBP, BMI, total cholesterol, HDL-C, LDL-C, triglycerides, individual cardiovascular risk score, Medi-Lite score), calculated in the whole sample

Risk factors (units of measurement)	Baseline (T_0_)	Follow-up (T_1_)	Δ Mean / Median [95%CI]	*p*-value
Waist circumference (cm)	89.02	87.97	1.25 [0.20; 2.70] *	0.09
Weight (kg)	75.22	73.95	1.27 [0.12; 2.40] *	**0.03**
Systolic Blood Pressure (mmHg)	133.34	120.12	13.22 [7.63; 18.81] *	**< 0.0001**
Diastolic Blood Pressure (mmHg)	86.97	75.78	9.5 [5.00; 15.00] °	**0.0001**
BMI (kg/m^2^)	26.99	26.52	0.41 [0.04; 0.81] °	**0.02**
Total Cholesterol (mg/dL)	211.39	203.24	11.5 [2.00; 20.00] °	**0.02**
HDL-C (mg/dL)	55.41	56.85	− 1.5 [-4.00; 0.5] °	0.15
LDL-C (mg/dL)	134.25	126.87	12.5 [2.5; 20.00] °	**0.01**
Triglycerides (mg/dL)	110.78	97.51	7.0 [5.00; 19.00] °	0.24
Individual cardiovascular risk score	5.10	3.61	0.8 [0.30; 1.60] °	**0.0009**
Medi-Lite score	11	12	− 2.5 [-3.50; 1.50] °	**0.0001**

* = **Δ Mean; ° = Δ Median**

DBP also showed significant reduction at T_1_ compared with T_0_, with a median variation of 9.50 mmHg (95%CI = 5.00–15.00; p = 0.0001). Regarding the lipid profile, at T_1_ compared with T_0_, a significant decrease in total cholesterol of 11.5 (95%CI = 2.00–20.00; p = 0.02) was recorded, and LDL cholesterol decrease of 12.5 (95%CI = 2.5–20.00; p = 0.01).

A significant reduction in individual cardiovascular risk score was detected 12 months after the start of the study with median variation of 0.8 (95%CI = 0.30–1.60; p = 0.0009).

Analysis of changes in Medi-Lite score showed a significant improvement of 2.5 (95%CI = 3.50–1.50; p = 0.0001) between T_0_ and T_1_ in adherence to the Mediterranean diet: the most remarkable changes were the increase in consumption of vegetables, with an increase of 19.5% of participants who recorded score 2 (> 2.5 portions per day) and the decrease in consumption of meat and sausages, corresponding to an increase of score 2 of 19.5% (< 1 portion per week) and the decrease of milk and dairy products, corresponding to an increase of score 2 of 17.1% (< 1 portion per week) (Table 3).

**Table 3:** Differences in percentages of respondents to the Medi-Lite questionnaire who obtained the scores (0, 1, 2) for each food group, calculated between T_0_ and T_1_

Food groups	D% (T_1_-T_0_) Score		
	0	1	2
Fruits	-2.4	-4.9	+ 7.3
Vegetables	-17.1	-2.4	+ 19.5
Beans and legumes	-34.1	+ 29.3	+ 4.9
Cereals	-2.4	-4.9	+ 7.3
Fish	-9.8	+ 7.3	+ 2.4
Fresh and cold cut meat	-12.2	-7.3	+ 19.5
Milk and dairy products	-4.9	-12.2	+ 17.1
Olive oil	0	0	0
Alcohol	0	+ 2.4	-2.4

Important differences emerged from the gender analysis; the most significant parameters’ variation were observed among women (Table 4). Women reduced BMI of 0.47 (95%CI = 0.021.10; p = 0.03), DBP of 12 mmHg (95%CI = 5.99–19.50; p = 0.001) and SBP of 16.90 mmHg (95%CI = 16.90-25.65; p = 0.0006) while men reduced DBP of 7.00 mmHg (95%CI = 0.00–17; p = 0.04) and SBP of 9.35 mmHg (95%CI = 2.09–16.60; p = 0.01). On the contrary, for weight and waist circumference, the highest variations between T_0_ and T_1_ were found in men, although they were not statistically significant. In particular, among men, the average weight was found to be 79.8 kg at T_0_ and 78.4 kg at T_1_ (mean difference = 1.4 kg). In contrast, women had an average weight of 70.9 kg at T_0_ and 69.7 kg at T_1_ (mean difference = 1.2 kg). Furthermore, the average waist circumference in men decreased from 92.2 cm at T_0_ to 90.5 cm at T_1_ (mean difference = 1.7 cm). In women, the average waist circumference was 85.9 cm at T_0_ and 85.1 cm at T_1_ (mean difference = 0.8 cm).

**Table 4:** Differences in means and median, between T0 and T1, of the considered risk factors (waist circumference, weight, SBP, DBP, BMI, total cholesterol, HDL-C, LDL-C, triglycerides, individual cardiovascular risk score, Medi-Lite score), calculated by gender

Risk factors (units of measurement)	Women		Men	
	Δ Mean / Median [95%CI]	*p*-value	Δ Mean / Median [95%CI]	*p*-value
Waist circumference (cm)	0.80 [-0.98; 2,58] *	0.36	1.70 [-0.74; 4,14] *	0.16
Weight (kg)	1.16 [0.13; 2.20] *	**0.02**	1.37 [-0.81; 3.56] *	0.20
Systolic Blood Pressure (mmHg)	16.90 [16.90; 25.65] *	**0.0006**	9.35 [2.09; 16.60] *	**0.01**
Diastolic Blood Pressure (mmHg)	12.00 [5.99; 19.50] °	**0.001**	7.00 [0.0004; 17] °	**0.04**
BMI (kg/m^2^)	0.47 [0.02; 1.10] °	**0.03**	0.27 [-0.26; 1.04] °	0.23
Cholesterol (mg/dL)	13.50 [2.00; 22.00] °	0.01	9.00 [-19.50; 25.00] °	0.39
HDL-C (mg/dL)	< 0,0001 [-3.00; 2.99] °	0.91	-3.00 [-6.4; 0.000061] °	0.05
LDL-C (mg/dL)	14.00 [4.00; 24.49] °	**0.01**	8.00 [-19.00; 24.50] °	0.34
Triglycerides (mg/dL)	7.00 [-4.99; 19.00] °	0.15	6.00 [-17.50; 35.50] °	0.57
Individual cardiovascular risk score	1.16 [0.33; 4.25] °	**0.002**	0.62 [-0.05; 1.35] °	0.08
Medi-Lite score	-2.50 [-3.00; -1.49] °	**0.002**	-2.50 [-4.00; -0.99] °	**0.01**

* = **Δ Mean; ° = Δ Median**

## Discussion

This study evaluates the impact of a multidisciplinary educational and training intervention on CV risk factors and patient adherence to MD. This study is part of a larger primary cardiovascular prevention project; [29]. but it is focused on assessing the role of MD and nutritional intervention in improving subjects’ dietary habits and hematochemical and anthropometric parameters.

Multidisciplinary educational interventions have been proved to influence and change people’s lifestyles. Indeed, the study’s results showed that the most relevant differences (p < 0.05) were recorded for SBP and DBP, total cholesterol and LDL-C, BMI, individual CV risk and Medi-Lite score. These changes could be due to the impact of the multidisciplinary educational intervention on the adoption of healthy lifestyles involving MD, physical activity, and smoking cessation, as reported by many studies [30, 31].

Specifically, in accordance with scientific evidence, the participant’s lifestyle change had positive impacts on the outcomes of the many parameters, such as weight, BMI reduction, [32, 33]. the variation of SBP and DBP [7, 34]. and the decrease in LDL-C and total cholesterol levels [35]. In addition, the improvement of MD adherence assessed thought Medi-Lite could be attributed to the educational intervention. This finding could also represent an innovative and original result since several studies on lifestyle interventions did not involve the MD, [36, 37]. but other kinds of diet. In particular, an increase in the consumption of fruit, vegetables, legumes, and fish was highlighted among the study participants, while that of fresh meat and cold cut meat decreased. These findings are encouraging, since people adhered to MD principles, and this suggests the effectiveness of the training intervention.

The variation of the anthropometric and haematochemical parameters, as well as the increase in MD adherence, suggests a reduction of the individual cardiovascular risk score, as found in our results. This finding agrees with what has been reported by several studies such as Gomez-Huelgas et al. that found the reduction of abdominal circumference (-0.4 ± 6 cm, p < 0.001), systolic blood pressure (-5.5 ± 15 mmHg; p = 0.004), diastolic blood pressure (-4.6 ± 10 mmHg; p < 0.001) and HDL-cholesterol (+ 4 ± 12 mg/dL; p = 0.05) after a lifestyle intervention [31, 38].

Another important consideration concerns the gender issue that highlighted the weight, BMI, LDL-C, and individual CV risk score significantly decreased only among female participants, in disagree with some other studies [39, 40]. In fact, Kent Lillian et al. found that the reduction of total cholesterol and LDL, BMI was better in men than in women. Moreover, although the variation of DBP and SBP was significantly decreased among both men and women, women recorded a greater reduction than men [39].

Similarly, Medi-Lite score significantly increased in both groups. Although most of the women in the sample were in the menopausal stage (average age 55.4 years), they had a greater reduction of CV risk score, if compared to men. However, since some studies suggest the potential underestimation of hypertension detection and cardiovascular risk categorization in women, further investigations would be appropriate to better understand this phenomenon [41–43]. Nevertheless, this is a promising finding in a primary prevention perspective, considering that menopause marks an increase in CV risk for women [44]. Therefore, the results suggest that adherence to the MD and a good level of physical activity are associated with a better cardio-metabolic profile and an improvement in menopausal symptoms, [45]. as also shown by Barrea et al. and Lombardo et al [46, 47].

Although the findings should be interpreted with caution, the main strength of this study is the comprehensive multidisciplinary approach employed by the research team [48, 49]. This program incorporated a variety of perspectives and approaches, including input from experts in different fields, and utilized a range of educational techniques to enhance the health status of participants. In fact, health promotion and primary prevention interventions play an essential role in the overall awareness of well-being and the multidisciplinary approach is able to ensure the patient a constant educational and clinical support, increasing compliance with preventive and therapeutic indications and improving the outcomes of the intervention. The results demonstrate the importance of multidisciplinary interventions in the prevention of cardiovascular diseases, supporting their implementation in primary prevention at the general population level. Therefore, the introduction of multidisciplinary intervention programs could be an effective strategy to improve the lifestyle of the population, reducing the burden on public health.

Despite the effectiveness in the reduction of CV risk and increase in MD adherence, the study shows some limitations: the sample size (41 subjects) is small and the observation period, lasting one year from November 2018 to November 2019, could be too short to assess long-term changes in lifestyles and CV risk. Furthermore, data regarding the use of drugs were incompletely collected, and we did not consider them usable and appropriate for the study. Moreover, the Medi-Lite, despite having been disclosed and validated in scientific literature, [28]. had gaps regarding the standard portions and frequencies. These are reported differently in the “Reference Intake Levels of nutrients and energy” (LARN – Italian acronym) published by SINU [20]. Finally, the final score ranges for assessing adherence to MD could be too wide (0–4 = Not adequate; 5–9 = Slightly adequate; 10–15 = Sufficiently adequate; 16–18 = Completely adequate), therefore improvements in individual Medi-Lite score could not always determine a category shift, thus flattening the results. Therefore, the Medi-Lite questionnaire could be not sensitive enough to detect small changes.

In this study, the role of the nutritional intervention in changing the subjects’ eating habits was evaluated. However, physical and psychological interventions were also included, so that the diet was not the only factor contributing to the final results. There are indeed many studies showing the benefits of a multidisciplinary approach to weight control programs, especially in the context of obesity, [50–52]. as also acknowledged by specfic guidelines [53]. In the context of our study, it is not possible to assess the specific impact of each intervention in changing lifestyle and CV risk reduction and it is hard to make conclusion for the roles of Mediterranean diet in the intervention.

Future prospects could be to extend the sample and continue the study over a longer timeframe, as well as the development of more specific data collection tools. Moreover, estimating the effectiveness that each specific intervention has on improving the cardiovascular health of people involved may also be appropriate.

## Conclusions

The findings of the study indicate that educational interventions can effectively enhance the health status of individuals. This is an important insight, as it suggests that targeted interventions aimed at promoting healthy behaviors and practices can be effective in improving public health outcomes.

Furthermore, the study suggests that a multidisciplinary approach can be particularly useful in enhancing the effectiveness of educational interventions. By bringing together experts from different fields a more comprehensive and integrated approach to primary prevention can be developed.

## Data Availability

The datasets used and/or analysed during the current study are available from the corresponding author on reasonable request. The data are not publicly available.

## List of abbreviations

CVDs: Cardiovascular diseases
DALYs: Disability-adjusted life years
BP: Blood pressure
SBP: Systolic blood pressure
DBP: Diastolic blood pressure
MD: Mediterranean diet
CREA: “Council for Agricultural Research and Agricultural Economics Analysis” (Italian acronym)
SINU: “Italian Society of Human Nutrition” (Italian acronym)
GP: General practitioner
BMI: Body Mass Index
FFQ: Food frequency questionnaire
LARN: “Reference Intake Levels of nutrients and energy” (Italian acronym)
